# A Computational Method for Systematic Comparison of Clinical Practice Guidelines

**DOI:** 10.1002/gin2.70071

**Published:** 2026-04-23

**Authors:** Kees C. W. J. Ebben, Astrid C. P. Swinkels, Cornelis D. de Kroon, Channa E. Schmeink, Wui‐Jin Koh, Olga L. van der Hel, Thijs van Vegchel, Michèle Thissen, Ignace H. J. T. de Hingh, Jurrian van der Werf

**Affiliations:** ^1^ Department of Research and Development Netherlands Comprehensive Cancer Organization (IKNL) Utrecht The Netherlands; ^2^ Department of Epidemiology GROW‐School for Oncology and Developmental Biology Maastricht University Maastricht The Netherlands; ^3^ Department of Obstetrics and Gynecology Leiden University Medical Center Leiden The Netherlands; ^4^ Department of Obstetrics and Gynecology Anna Hospital Geldrop The Netherlands; ^5^ National Comprehensive Cancer Network Philadelphia USA; ^6^ Department of Surgical Oncology Catharina Hospital Eindhoven The Netherlands

**Keywords:** clinical decision support, computable guidelines, harmonization, rule‐based knowledge representations, semantic interoperability, standardization

## Abstract

**Introduction:**

Clinical practice guidelines are essential in improving oncology healthcare by translating scientific evidence into practice. Evaluating and comparing guidelines for the same medical condition across organizations and countries aids in assessing their validity. However, inconsistencies in their format and the topics covered hamper large‐scale efficient guideline comparisons. This study applies a technical format to guidelines from two regions and introduces a computational method for systematic guideline comparison.

**Methods:**

For every decision‐making moment identified in the Dutch and US guidelines for endometrial cancer clinical decision trees (CDTs) were developed. These were organized into coherent care pathways and classified according to an oncological reference model. Subsequently, calculations and content comparisons were performed on the revealed concepts at a semantic level. Lastly, recommendations were compared across the subpopulation(s) defined within each CDT.

**Results:**

The method identified similarities, relations and differences at various levels between both guidelines. Analyses yielded 17 CDTs, 33 population characteristics and 75 interventions for the Dutch guideline, while the corresponding figures for the US guideline were 20, 61 and 111, respectively. Within the CDTs, 11 matching subpopulations were identified, of which 2 had identical recommendations, 5 had related recommendations and 4 showed mismatched recommendations.

**Discussion:**

The current computational approach supports structured comparisons in research settings and allows for application in clinical practice via a developed dashboard prototype. Computational guideline representations reveal gaps, differences and similarities across multiple levels. The study underscores the potential for increased utility of computable guidelines, with the developed method also applicable to various other rule‐based instruments.

## Summary

- A computable representation of guidelines enables objective and reproducible comparison of their content and decision logic.
- Observed differences between guidelines are largely attributable to variation in terminology and granularity, rather than underlying evidence.
- Explicit structuring of guideline components allows systematic identification of inconsistencies and alignment gaps.
- The approach provides a basis for integration into decision support and more efficient guideline updating.

## 1 Introduction

Clinical practice guidelines play a pivotal role in enhancing the quality of healthcare delivery [1]. These guidelines, based on systematic literature reviews, are fundamental in translating scientific evidence into clinical practice. However, the process of developing guidelines encompasses myriad methods, often leading to questions surrounding their validity [2]. Despite the accessibility of empirical evidence for guideline developers, disparities persist in guidelines across organizations and countries, even when grounded in similar evidence‐based recommendations and population characteristics [3]. The validity of guidelines is of high concern, as they serve as the backbone for integrating scientific evidence into clinical decision‐making [4]. Continuous updating is essential, but the methodologies for this process are often unclear [5]. Moreover, the development and updating of guidelines demand substantial resources and labour, with many organizations engaged in this endeavour lacking a formalized process for determining when a guideline becomes outdated [6]. Some international guidelines are updated at short intervals, making them useful reference points for evaluating the currency and relevance of national guidelines [7]. However, such comparisons are only meaningful when both sets of guidelines are of high quality, developed within the same time frame and reflect the most up‐to‐date evidence.

Efforts to compare guidelines, particularly in oncology, have been extensive [8, 9, 10, 11, 12, 13, 14, 15]. However, many comparisons focus on interventions while overlooking differences in patient populations. Discrepancies often stem from guideline development methodologies that prioritize specific clinical questions in isolation from the broader care continuum [16]. Conflicting recommendations highlight the scarcity of high‐level evidence and reflect regional healthcare variations and cultural distinctions [17]. Guideline comparisons offer valuable insights into clinical options and help ensure equity in treatment access across countries. Moreover, systematically comparing guideline content supports their adoption and adaptation [18]. Guideline differences may also influence variations in disease outcomes between countries, underscoring the need for rigorous analysis.

A notable example of guideline comparison is described by Alper and colleagues, who identified key structural elements, such as similarity in population, intervention, direction and strength, for comparing recommendations across guidelines and point‐of‐care resources [19]. Another promising avenue for a more systematic and computational approach to comparing guidelines was presented by Song and colleagues, who employed algorithmic flowcharts to compare 18 guidelines pertaining to hepatocellular carcinoma from Asian, European and US sources, marking a stride towards leveraging health informatics to support guideline comparison and implementation [20]. The emergence of computable guidelines opens up new possibilities for in‐depth analysis of the guideline key elements [7]. As emphasized by Alper, expressing guidelines in a machine‐interpretable format reduces redundant tasks for developers and facilitates more efficient updates [21]. This aligns with our approach, where such representations enable systematic comparison of recommendations across guidelines. Hendriks and colleagues pioneered a method for transforming guideline knowledge into a computable format, breaking down narrative text into clinical decision trees (CDTs) structured around decision points in clinical care pathways [22]. While CDTs remain human‐readable, they also facilitate computer‐assisted evaluation of the underlying algorithms. Converting knowledge from guidelines, and other formatted sources, into uniform, computer‐interpretable representations supports a wide range of possible analyses. Computational guidelines may have a function in the development of guidelines (comparability), clinical implementation of guidelines (decision support systems) and monitoring of guideline use in clinical practice (research).

In the present study, we build upon this method by comparing two guidelines addressing the same disease. Conducted within the Alertness project and funded by ZonMw [23], this study aligns with the project's goal of enhancing guideline development. Consequently, the methodology was applied to guidelines for endometrial cancer issued by the Dutch Society for Oncological Gynecology (NVOG) in the Netherlands and the National Comprehensive Cancer Network (NCCN) in the United States. The primary objective of our research is to demonstrate a systematic approach to guideline comparison using CDTs. Secondary objectives encompass the identification of similarities and disparities in counts and content, regarding (a) decision points, represented by CDTs, (b) CDT complexity, (c) population characteristics and (d) recommendations. In doing so, we aim to contribute to the refinement and standardization of guideline comparison methods, enhancing the utility and reliability of clinical practice guidelines in healthcare decision‐making. Ultimately, this would help to minimize divergence in disease outcomes resulting from discrepancies in guidelines.

## 2 Methods

### 2.1 Guideline Selection for Comparison

The methodology employed in this study was applied to analyze two distinct national guidelines for endometrial cancer. The guidelines under consideration are the Dutch guideline, known as the ‘Richtlijn Endometrium carcinoom’ (version 2022) [24], and the NCCN guideline ‘Uterine Neoplasms’ (version 1.2022) [25], specifically the ‘Endometrial Cancer’ section. The Dutch guideline, under the governance of the NVOG, has been crafted in a narrative format and is generally organized by type of intervention. In contrast, the NCCN guideline is predominantly presented as decision algorithms aligned with clinical processes, and is supplemented by footnotes and addenda to provide in‐depth insights into specific areas.

### 2.2 Algorithmic Knowledge Representation

**Figure 1:**
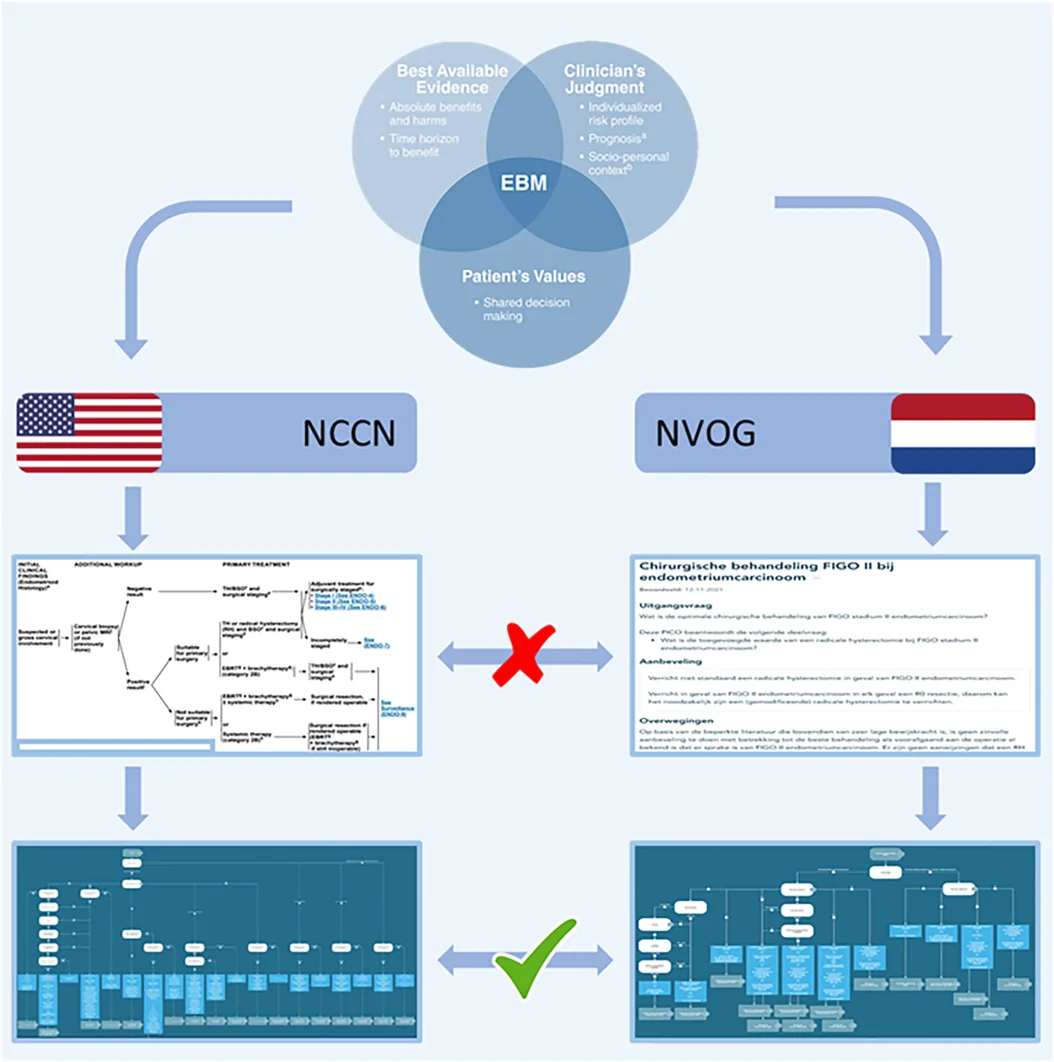
Illustrative overview of clinical practice guideline and clinical decision tree development. Clinical practice guidelines are developed by various organizations following evidence‐based medicine principles [26] resulting in diverse formats and visual presentations. This figure highlights the structural variety of these guidelines for illustrative purposes only; the content is not intended to be readable. Transforming guidelines into standardized clinical decision trees facilitates computational analysis and structured comparison of their content.

To enable a robust and meaningful computational comparison, we have undertaken the task of parsing the content from both guidelines into a standardized and identical format. This harmonization process is illustrated in Figure 1, and it forms the cornerstone of our analytical approach. In line with the methodology detailed by Hendriks et al. [22], our study involves the transformation of the respective guidelines into CDTs to comprehensively pinpoint all relevant concepts essential for informed decision‐making in the context of endometrial cancer. These concepts encompass all population characteristics, as well as interventions revealed by the guideline. Subsequently, these identified concepts are organized into a shared vocabulary.

### 2.3 Data Analysis

**Figure 2:**
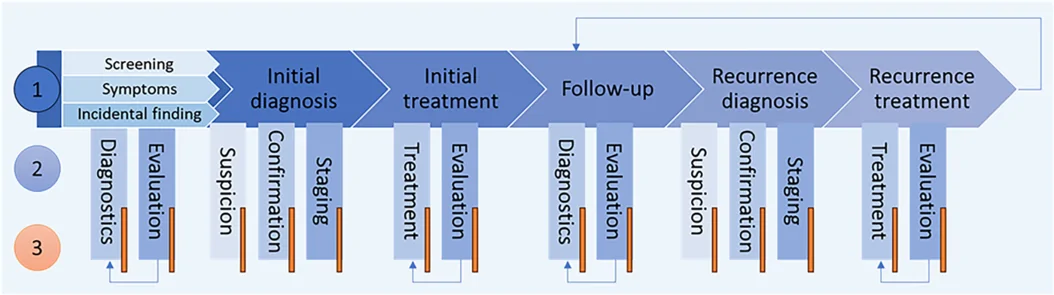
The generic oncological process. The generic oncological clinical process serves as a reference model, offering a standardized depiction of the highest‐level oncological care process. The universally applicable process steps (1) provide a generic framework for all oncological conditions. Each step can be detailed into smaller standard steps (2), representing one or more decision points, ideally equipped with a guideline recommendation (3).

Uniform representations of guidelines in the form of CDTs enable a structured evaluation of their content. For a thorough content comparison, we utilize a reference model that comprises a systematically structured representation of the oncological care process. These process steps are universally applicable to all oncological conditions, providing a generic framework. As presented in Figure 2, all content comparisons between guidelines are conducted for each ‘generic oncological clinical process step’. The oncological care pathway is stratified according to this process since every phase has its unique interpretation and application of clinical concepts. Our comparative analysis unfolds through several distinct steps:

1. *Calculations*:

a) First, we performed a quantitative comparison by calculating the number of CDTs and their constituent concepts, which encompass population characteristics, and diagnostic and therapeutic interventions. We analyzed the number of population characteristics per CDT, recognizing that these characteristics may occur multiple times within a CDT.
b) Next, complexity scores are calculated for all CDTs within each guideline. CDT complexity is defined as the sum of four components: (I) the total number of population characteristics included in the CDT; (II) the number of unique population characteristics (as individual characteristics may appear more than once within a single CDT); (III) the number of subpopulations, defined as patient groups characterized by a specific combination of population characteristics that the guideline explicitly identifies as relevant to a particular recommendation (in the CDT representation, each subpopulation corresponds to a unique path from top to bottom); and (IV) the maximum number of population characteristics used to define a subpopulation, represented by the longest path in the CDT [27]. The average complexity scores per guideline are then reported.

2. *Content representation evaluation*:
  During modelling guidelines into a computable format, recommendations relevant to the same point in the care pathway are grouped into a single CDT. Each CDT represents a distinct decision moment, providing recommendations for multiple subpopulations.

a) First, we assessed if similar decision moments were present: (I) in both guidelines, (II) as related CDTs across guidelines or (III) in just one guideline. Relationship was assessed based on the presence of recommendations relevant to comparable moments along the care pathway. This analysis allowed us to identify corresponding CDTs, facilitating a basis for subsequent comparison.
b) We performed a comparison of the concepts, including CDTs, population characteristics and interventions, between the two guidelines. In the textual comparison, concept terms were categorized as identical, related or no match.
  - *Identical Match*—Concepts are exact synonyms.
  ∘ Example: the term ‘grade’ is used in both guidelines.
  - *Related Match*—Concepts are related but not identical.
  ∘ Example: CT abdomen and PET‐CT neck/chest/abdomen/pelvis.
  - *No Match*—Concepts were present in only one guideline.
  ∘ Example: A recommendation available in the NVOG guideline but absent in the NCCN guideline, or vice versa.
  A concept from one guideline could also relate to multiple concepts from the other. A more detailed and extended elaboration of the applied semantic analyses can be found in Supporting Information S1 and S2: Supplements 1 and 2.

3. *Subpopulations and recommendations comparison*
  Ultimately, we compared recommendations for identified identical and related subpopulations between guidelines. *As defined above, each subpopulation corresponds to a unique path through the CDT*. Analyses are based on the mandatory concepts as presented in the following equation, representing a guideline recommendation:
R=𝚺(I+D+S)±C
  R = Recommendation; I = Intervention; D = Direction; S = Strength; C = ‘Other considerations’.
  Equation 1. Comparation of a clinical practice guideline recommendation.
  The metadata descriptors ‘direction’ (perform vs. abstain) and ‘strength’ (weak vs. strong) provided with corresponding interventions per guideline were taken into account in the recommendation comparison. ‘Other considerations’ are not mandatory and were not analyzed due to high variability within and between guidelines.

### 2.4 Visualization of Results

Analyses were performed offline, using Stata version 17 software (StataCorp LLC, College Station, TX, USA). The results of the developed methodology are presented in a prototype of a web‐based dashboard, which enables interactive exploration and comparison of the analyses across the above‐mentioned criteria.

In addition to the analyses presented in the main text, supplementary analyses were conducted using SNOMED CT. The methodology for these analyses is detailed in Supporting Information S1: Supplement 1, while Supporting Information S2: Supplement 2 presents the resulting data in tabular form, providing interested readers with full transparency of the additional findings.

## 3 Results

### 3.1 Guideline Selection for Comparison

**Figure 3:**
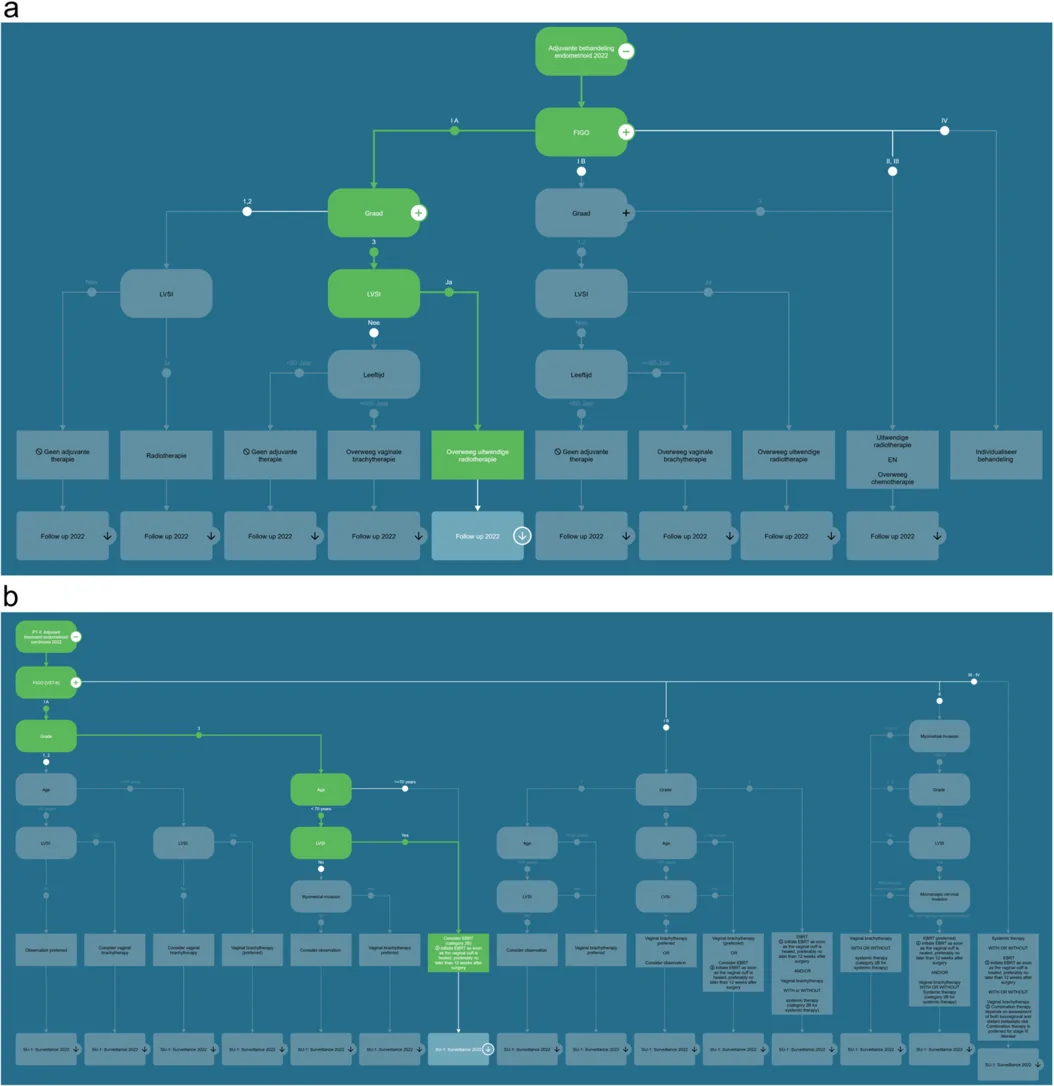
Clinical decision trees (CDTs) for adjuvant treatment of endometrioid carcinoma according to the NVOG (a) and NCCN (b) guidelines. The NCCN CDT includes one additional population characteristic (myometrial invasion) compared with the NVOG CDT. Therefore, these CDTs were marked as related for further analysis. In both panels, the green‐highlighted decision path illustrates a single subpopulation terminating in its associated recommendation at the bottom (square panel). In this example, the subpopulations of this particular recommendation are different, with ‘age’ additionally present in the NCCN path. The highlighted recommendations for this subpopulation are concordant, with both guidelines recommending external beam radiotherapy (EBRT), with the NCCN recommendation supplemented by additional considerations. Therefore, the recommendation for this subpopulation is classified as a ‘related match’ (as opposed to identical or no match).

In the initial phase of our study, we parsed each decision‐point from both guidelines individually and proceeded to connect these into a cohesive care pathway. Subsequently, each decision point and its underlying characteristics were modelled to reflect the intent of the original guideline. To ensure the accuracy of this representation, all CDTs were validated and approved by the respective guideline holders of both the NVOG and NCCN guidelines. Within the domain of oncology, we identified and categorized six ‘generic oncological clinical process steps’, which encompass screening, initial diagnostics, initial treatment, follow‐up, recurrence diagnostics and recurrence treatment. Each distinct CDT was classified as an element within one of these defined process steps. Figure 3 depicts the CDTs of both guidelines regarding ‘adjuvant treatment’ in the initial treatment phase and has been included to provide a clear illustration of the comparative structure of the guidelines.

### 3.2 Calculations

The NVOG guideline was parsed into 17 CDTs, while the NCCN guideline yielded 20 CDTs. In total, we identified 33 population characteristics, as well as 65 recommended interventions in the NVOG guidelines, and 59 population characteristics, along with 109 recommended interventions in the NCCN guideline. Results considering the generic oncological process steps are presented in Table 1. Neither guideline contained recommendations regarding screening. The CDTs based on the NCCN guideline exhibited a notably higher mean complexity score (Table 2).

**Table 1:** Guideline concepts.

GOPS	NVOG CDTs	No. PCs	No. recommendations	NCCN CDTs	No. PCs	No. recommendations
*Initial diagnosis*						
	Initial diagnostics	0	1	Initial evaluation	0	9
	Additional diagnostics	1	1	Initial work‐up imaging	4	5
	Staging diagnostics	3	4	Additional chest imaging	3	3
				Additional diagnostics	2	4
				Additional diagnostics for incidental findings	1	2
Total		4	6		10	23
*Initial treatment*						
	Primary treatment	8	12	Primary treatment	9	19
	Staging evaluation	1	2	Staging evaluation	1	2
	Adjuvant treatment endometrioid adenocarcinoma	4	6	Adjuvant treatment endometrioid adenocarcinoma	6	5
	Adjuvant treatment sereus/clear cell carcinoma	0	1	Protocol after endometrial evaluation (fertility sparing) at 3–6 months	1	5
	Policy after hormonal therapy	0	1	Re‐evaluation for surgery endometrioid carcinoma	3	5
	Primary treatment; child wish	1	1	Protocol after incomplete staging	5	7
	Continuation treatment; child wish	3	4	Evaluation of imaging after incomplete staging	1	3
	Conception phase; child wish	1	2	Adjuvant treatment high risk carcinoma	5	4
				Re‐evaluation for surgery high risk carcinoma	4	6
				Protocol after endometrial evaluation (fertility sparing) every 6 months	4	7
Total		18	29		39	63
*Follow‐up*						
	Follow‐up	1	3	Surveillance	1	4
	Follow‐up; during conception phase	2	3			
	Continuation follow‐up; during conception phase	1	2			
Total		4	8		1	4
*Recurrence diagnosis*						
	Recurrence diagnostics	1	2	Recurrence diagnostics	1	5
Total		1	2		1	5
*Recurrence treatment*						
	Recurrence treatment	6	8	Therapy for relapse	5	7
	Policy after hormonal therapy; follow‐up	0	1	Additional therapy for locoregional relapse	2	3
				Protocol after therapy for relapse	1	4
Total		6	9		8	14
Grand total		33	54		59	109

*Note:* Unique number of concepts per generic oncological process step and clinical decision tree.

Abbreviations: CDT = clinical decision tree; GOPS = generic oncological process steps; PC = population characteristics.

**Table 2:** Clinical decision tree complexity.

NVOG CDTs	No. population characteristics	No. subpopulations	The highest number of population characteristics used to define a subpopulation within a CDT	Unique count of population characteristics within a CDT	Total	NCCN CDTs	No. subpopulations	No. subpopulations	The highest number of population characteristics used to define a subpopulation within a CDT	Unique count of population characteristics within a CDT	Total
Initial diagnostics	0	1	1	0	2	Initial evaluation	0	1	1	0	2
Additional diagnostics	1	2	2	1	6	Additional diagnostics	2	8	3	2	15
Staging diagnostics	3	4	4	3	14	Initial work‐up imaging	4	5	4	4	17
Primary treatment	9	11	7	8	35	Additional chest imaging	4	6	3	3	16
Staging evaluation	1	2	2	1	6	Additional diagnostics for incidental findings	1	3	2	1	7
Adjuvant treatment endometrioid adenocarcinoma	8	10	5	5	28	Primary treatment	16	19	8	9	52
Continuation treatment; fertility preserving	3	4	4	3	14	Staging evaluation	1	2	2	1	6
Adjuvant treatment sereus/clear cell carcinoma	0	1	1	0	2	Adjuvant treatment endometrioid adenocarcinoma	17	15	6	7	45
Policy after hormonal therapy	0	1	1	0	2	Protocol after endometrial evaluation (fertility sparing) every 3 months	1	2	2	1	6
Primary treatment; fertility preserving	1	2	2	1	6	Re‐evaluation for surgery endometrioid carcinoma	3	5	3	3	14
Conception; fertility preserving	1	2	2	1	6	Protocol after incomplete staging	10	11	6	5	32
Follow‐up	1	3	2	1	7	Evaluation of imaging after incomplete staging	1	2	2	1	6
Follow‐up; during conception phase	1	2	2	1	6	Adjuvant treatment high risk carcinoma	9	10	6	5	30
Continuation follow‐up; during conception phase	2	3	3	2	10	Re‐evaluation for surgery high risk carcinoma	4	6	4	4	18
Recurrence diagnostics	1	2	2	1	6	Protocol after endometrial evaluation (fertility sparing) every 6 months	4	4	5	4	17
Recurrence Treatment	8	10	5	6	29	Surveillance	1	3	2	1	7
Policy after hormonal therapy; recurrence	0	1	1	0	2	Recurrence diagnostics	1	2	2	1	6
						Therapy for relapse	6	5	5	6	22
						Additional therapy for locoregional relapse	2	4	3	2	11
						Protocol after therapy for relapse	1	3	2	1	7
	Total				181		Total				336
	Mean				10.6		Mean				16.8

*Note:* Clinical decision trees complexity scores based on number of population characteristics (represented by CDT nodes), number of subpopulations (represented by a path within a single CDT from top till bottom), the highest number of population characteristics used to define a subpopulation within a CDT (represented by the longest CDT path), and the unique count of population characteristics within a CDT per clinical decision tree and the mean total score.

### 3.3 Content Representation Evaluation

In the initial assessment, 5, 5 and 17 CDTs were classified as ‘identical’, ‘related match’ and ‘no match’, respectively. For population characteristics, the assessment yielded classifications of 10 for ‘identical’, 16 for ‘related match’ and 46 for ‘no match’. Regarding interventions, the assessment yielded classifications of 20 for ‘identical’, 39 for ‘related match’ and 47 for ‘no match’. The substantive findings derived from these analyses are presented in Supporting Information S1: Supplement 1.

### 3.4 Subpopulations and Recommendations Comparison

As shown in Table 3, the 10 identical or related CDTs contained a total of 11 subpopulations that were defined identically in both sets of guidelines. In two instances, the recommendations for these subpopulations were identical. In five instances, these were related, and in four cases, they differed substantially. The related recommendations often varied in terms of level of detail or terminology. For example, one guideline might recommend ‘consider chemotherapy’, while the other uses ‘systemic therapy’, reflecting a difference in detail level of the intervention and in recommendation strength. In other instances, similar interventions were described using different terminology and detail level. For example, ‘anamnesis AND gynecological physical exam’ in the Dutch guideline is related to ‘history AND physical’ in the US guideline. However, there were also clear cases of substantive disagreement between the guidelines. One such example is a Dutch recommendation for ‘EBRT AND consider chemotherapy’, whereas the US guideline for the same subpopulation recommends only to ‘observe’.

**Table 3:** Recommendation comparison for identical and related subpopulations.

Identical and related CDTs	Number of identical subpopulations	Subpopulation definition	Recommendation NVOG	Recommendation NCCN	Recommendation classification (identical, related, different)
Initial diagnostics (NVOG) AND Initial evaluation (NCCN)^a^	1	NA	– Anamnesis AND Gynecological physical exam AND Transvaginal ultrasound^b^	– History AND Physical AND Complete blood count AND Endometrial biopsy AND Genetic evaluation of tumour AND Evaluation for inherited cancer AND Consider liver function test AND Consider renal function tests AND Consider chemistry profile^b^	Related
Additional diagnostics	0				
Primary treatment	0				
Evaluation of staging	2	1. Result staging = complete	– Adjuvant treatment^c^	– Adjuvant treatment^c^	Identical
		2. Result staging = Incomplete	– Restaging	NA: Separate algorithm for further policy	NA
Adjuvant treatment endometrioid carcinoma	5	1. FIGO‐stage = IB AND Grade = 1 AND Age < 60 AND LVSI = No	– No adjuvant treatment^b^	– Consider observation^b^	Different
		2. FIGO‐stage = IB AND Grade = 2 AND Age < 60 AND LVSI =No	– No adjuvant treatment^b^	– Consider observation^b^	Different
		3. FIGO‐stage = IB AND Grade = 3	– EBRT^c^; OR	– EBRT^c^; OR	Identical and related
			– Consider chemotherapy	– EBRT AND Vaginal brachytherapy; OR	
				– EBRT AND Vaginal brachytherapy AND Systemic therapy	
		4. FIGO‐stage = III	EBRT AND Consider chemotherapy^b^	– Systemic therapy; OR	Related
				– Systemic therapy AND EBRT^b^; OR	
				– Systemic therapy AND EBRT AND Vaginal brachytherapy	
		5. FIGO‐stage = IV	Individualize treatment	– Systemic therapy; OR	Different
				– Systemic therapy AND EBRT; OR	
				– Systemic therapy AND EBRT AND Vaginal brachytherapy	
Adjuvant treatment sereus/clear cell (NVOG) AND Adjuvant high‐risk (NCCN)^a^	2	1. Morphology = Sereus carcinoma AND Result primary surgery = No residual	EBRT AND Consider chemotherapy	– Observe	Different
		2. Morphology = Clear cell carcinoma AND Result primary surgery = No residual	EBRT AND Consider chemotherapy	– Observe	Different
Child wish and continued treatment (NVOG) AND Protocol after endometrial evaluation (fertility sparing) every 3 months (NCCN)^a^	0				
Follow‐up (NVOG) AND Surveillance (NCCN)^a^	1	1. Years in surveillance = 3–5 years	Outpatient visit every 12 months AND Physical exam AND Gynecologic physical exam AND Counselling^b^	– Outpatient visit every 6 months AND Physical exam AND CA‐125 AND Imaging AND Patient education^b^	Related
Recurrence diagnostics	0				
Recurrence treatment (NVOG) AND Therapy for relapse (NCCN)^a^	0				

*Note:* Comparison of recommendations in identical and related subpopulations. Unless stated otherwise, these recommendations are strong and should be implemented: ‘Do’.

Abbreviations: CDT = clinical decision tree; EBRT = external beam radiotherapy; LVSI = lympho vascular space invasion; NA = not applicable.

a Related clinical decision trees.

^b^ Related recommendations.

^c^ Identical recommended intervention.

**Figure 4:**
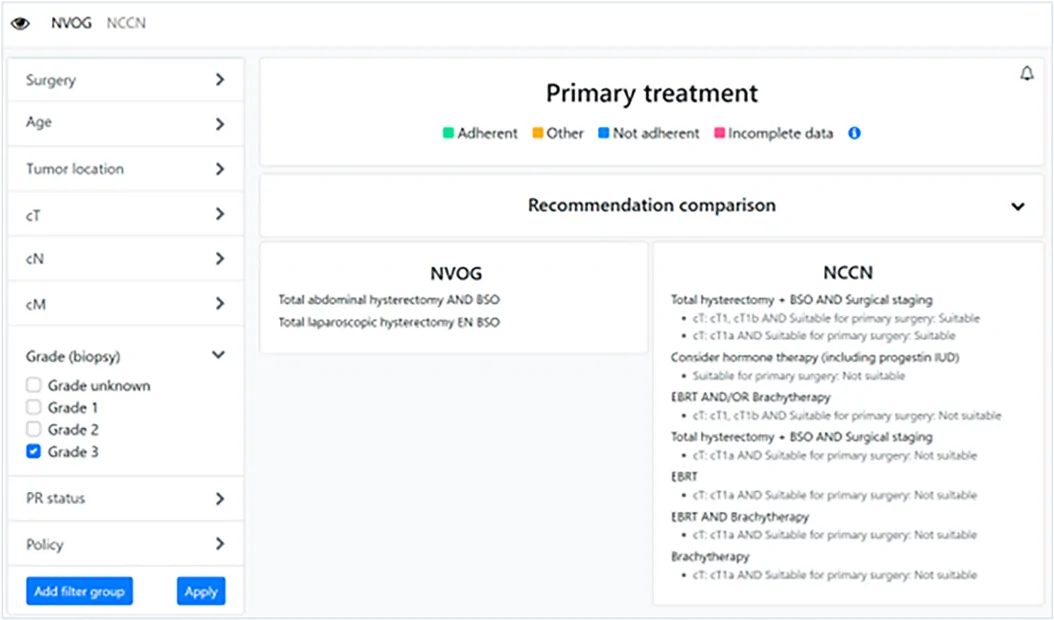
Alertness dashboard screenshot. The Alertness dashboard prototype incorporates a left‐side panel for streamlined population filtering. On the right, recommendations from both NVOG and NCCN endometrial cancer guidelines are presented based on applied filters. In this screenshot, the following filter was activated: Primary treatment; AND cT = 1a/1b/1; AND cN = 0; AND cM = 0; AND Grade = 3. Relevant recommendations from both guidelines are presented side by side. The NCCN guideline improves specificity by furnishing extra population characteristics (listed in bullet points), further fine‐tuning subpopulation specifications for each recommendation.

In addition, a prototype dashboard has been developed in which recommendations from the respective guidelines can be shown for each combination of population characteristics (Figure 4).

## 4 Discussion

### 4.1 Summary of the Main Findings

In this study, a novel methodology for the computational comparison of clinical practice guidelines was applied. The computational approach enabled analysis of two national guidelines in a structured manner, making their similarities and differences more objectively identifiable through explicit representation of populations, decision criteria and recommendations. This structured comparison allowed guideline content to be examined at the level of formally defined components rather than relying on narrative interpretation.

Numerous guideline comparison studies in oncology have focused on identifying similarities, differences and areas of controversy across guidelines [9, 10, 11, 12, 14, 15]. Our results show that direct comparability between recommendations was limited, despite both guidelines addressing the same disease domain and being based on largely overlapping bodies of evidence. Importantly, we observed that comparability improved when population characteristics and intervention definitions were explicitly aligned, resulting in clearer identification of whether recommendations addressed the same clinical scenario. The improvement therefore lies in reducing ambiguity and subjectivity in comparison, rather than in convergence of the recommendations themselves.

### 4.2 Examples of Insights Gained From the Findings

The most notable finding of this study was the identification of distinct subpopulations and recommendations in each guideline, resulting in a limited number of direct matches. This divergence occurred despite similarities in the underlying evidence base. When guidelines were represented computationally, it became apparent that differences arose primarily from how evidence was operationalized into population characteristics and intervention descriptions, rather than from fundamentally different interpretations of evidence.

An example of this variation is the level of specificity used to describe recommended interventions. One guideline referred to ‘hormone therapy’, whereas the other specified ‘tamoxifen’ for a comparable clinical context. While such terminology may be readily interpreted by clinicians in practice, the computational representation treated these as distinct intervention entities. As a result, recommendations that were clinically related could not be directly matched, illustrating how heterogeneous terminology creates concrete barriers to structured comparison.

Similarly, differences in granularity influenced comparability. Broad intervention categories such as ‘systemic therapy’ encompassed multiple treatment modalities in one guideline, whereas the other guideline distinguished these modalities explicitly. These inconsistencies limited the ability to represent metadata descriptors as structured elements, despite their relevance for understanding how recommendations are formulated and applied. Rather than asserting the general importance of standardization, our findings demonstrate that failures in terminological and structural alignment manifest as identifiable gaps when guidelines are represented computationally.

Another insight was the higher number of population characteristics and recommended interventions in the NCCN guideline compared with the NVOG guideline, resulting in a higher mean complexity. This reflects a more granular exploration of subpopulations and a broader set of explicitly defined interventions. These differences were particularly evident in the initial treatment phase and could be quantified through the computational representation.

### 4.3 Strengths and Limitations in the Context of Existing Literature

Guideline comparison studies have traditionally relied on narrative or tabular approaches to identify similarities and differences between guidelines [9, 10, 11, 12, 14, 15]. While informative, these methods depend heavily on expert interpretation and implicit assumptions regarding equivalence between populations and interventions. A key strength of the present study is that it extends these approaches by enabling rule‐based, reproducible comparison of explicitly defined guideline components.

By formalizing populations and interventions, the computational approach reduces subjectivity and makes structural differences visible. This allows guideline methodologists to distinguish between differences arising from representational choices and those reflecting substantive variation in recommendations. In this sense, the method complements existing guideline comparison approaches rather than replacing them.

Several limitations should be acknowledged. The primary aim of this study was to develop and apply a method for computational guideline comparison. We did not analyze the scientific references underlying the guidelines, nor did we evaluate associations between guideline differences and clinical outcomes. Furthermore, transforming narrative guidelines into CDTs is labour‐intensive, and observed differences may partly reflect national guideline development procedures rather than content alone. These limitations are consistent with other methodological comparison studies and should be considered when interpreting the findings.

### 4.4 Implications, Applications and Next Steps

The findings of this study suggest that computational guideline comparison could facilitate more transparent and systematic evaluation of guideline alignment by making population definitions, intervention scope and decision logic explicit. Such transparency may help clarify whether apparent differences between guidelines arise from evidence interpretation or from structural and semantic variation.

It is appropriate to note that this approach may support future investigations into whether structural differences between guidelines are associated with variation in clinical practice or healthcare outcomes across settings, although this was not assessed in the present study. Importantly, the current results demonstrate how similarities and differences can be identified, but do not show how these findings should be operationalized in guideline development, updating or implementation.

In conclusion, this study demonstrates that computational representation of clinical practice guidelines enables more objective identification of similarities and differences by explicitly structuring populations, decision criteria and interventions. The findings show that limited comparability between guidelines often arises from differences in terminology and granularity rather than from evidence itself. For guideline methodologists, this approach provides a complementary method to existing comparison techniques by revealing structural and semantic mismatches that are otherwise difficult to detect, thereby supporting more rigorous evaluation of guideline variation and alignment.

## 5 Conclusion

In the current study, a novel methodology for a computational comparison of clinical practice guidelines was introduced, facilitating a comprehensive assessment of their similarities and differences within the same disease domain. In the context of evolving medical knowledge, healthcare advancements and the dynamic needs of the population of interest, we argue that it is imperative to consider the timely updating of clinical practice guidelines.

This study emphasizes the critical importance of maintaining guidelines that align with the best available evidence, accommodating shifts in population characteristics and staying up‐to‐date with scientific advances and clinical practices to deliver optimal care for the population of interest. Furthermore, high‐quality comparisons of guidelines from various regions are relevant for healthcare providers and decision‐makers to effectively provide the specific needs of patients and uphold the highest standards of care in the field, irrespective of geographic disparities. Ultimately, the versatility and usability of computational guidelines, and other rule‐based knowledge representations, hold vast potential to advance healthcare in a future‐ready manner.

## Author Contributions

**Kees C. W. J. Ebben:** conceptualization, investigation, funding acquisition, writing – original draft, methodology, validation, visualization, writing – review and editing, software, formal analysis, project administration, data curation, supervision, resources. **Astrid C. P. Swinkels:** methodology, validation, writing – review and editing, formal analysis, visualization. **Cornelis D. de Kroon:** conceptualization, funding acquisition, writing – review and editing, methodology, validation. **Channa E. Schmeink:** conceptualization, funding acquisition, methodology, writing – review and editing, validation. **Wui‐Jin Koh:** conceptualization, methodology, writing – review and editing. **Olga L. van der Hel:** conceptualization, methodology, validation, writing – review and editing. **Thijs van Vegchel:** software, conceptualization, funding acquisition, writing – review and editing, methodology, visualization. **Mechèle Thissen:** funding acquisition, writing – review and editing. **Ignace H. J. T. de Hingh:** supervision, methodology, writing – review and editing. **Jurrian van der Werf:** funding acquisition, conceptualization, investigation, writing – original draft, methodology, validation, writing – review and editing, formal analysis, supervision, resources.

## Conflicts of Interest

The authors declare no conflicts of interest.

## Supporting information


Supporting File 1

Supporting File 2

## Data Availability

This study did not involve any data from individuals. The analyses were conducted using publicly available clinical practice guidelines. No personal or patient data were collected, used or analyzed.

## References

[1] R. Graham , M. Mancher , D. Miller , S. Greenfield , and E. Steinberg , Clinical Practice Guidelines We Can Trust (Institute of Medicine, National Academies Press, 2011), 300. 24983061

[2] M. B. Eriksen and T. F. Frandsen , “The Impact of Patient, Intervention, Comparison, Outcome (PICO) as a Search Strategy Tool on Literature Search Quality: A Systematic Review,” Journal of the Medical Library Association 106 (2018): 420–431. 30271283 10.5195/jmla.2018.345 PMC6148624

[3] M. B. Harrison , F. Legare , I. D. Graham , and B. Fervers , “Adapting Clinical Practice Guidelines to Local Context and Assessing Barriers to Their Use,” Canadian Medical Association Journal 182, no. 2 (2010): E78–E84. 19969563 10.1503/cmaj.081232 PMC2817341

[4] L. M. García , A. J. Sanabria , E. G. Álvarez , et al., “The Validity of Recommendations From Clinical Guidelines: A Survival Analysis,” Canadian Medical Association Journal 186, no. 16 (2014): 1211–1219. 25200758 10.1503/cmaj.140547 PMC4216254

[5] R. W. Vernooij , A. J. Sanabria , I. Solà , P. Alonso‐Coello , and L. Martínez García , “Guidance for Updating Clinical Practice Guidelines: A Systematic Review of Methodological Handbooks,” Implementation Science 9 (2014): 3. 24383701 10.1186/1748-5908-9-3 PMC3904688

[6] P. Alonso‐Coello , L. Martínez García , G. J. M. Carrasco , I. Solà , S. Qureshi , and J. S. Burgers , “The Updating of Clinical Practice Guidelines: Insights From an International Survey,” *Implementation Science* 6 (2011): 1–8. 10.1186/1748-5908-6-107 PMC3191352 21914177

[7] M. Azarpira , A. Redjdal , J. Bouaud , and B. Seroussi , “Methods Used to Compare Narrative Clinical Practice Guidelines: A Scoping Review,” Studies in Health and Technology Informatics 295 (2022): 304–307. 10.3233/SHTI220723 35773869

[8] M. Ballester , S. Bendifallah , and E. Daraï , “European Guidelines (ESMO‐ESGO‐ESTRO Consensus Conference) for the Management of Endometrial Cancer,” Bulletin du Cancer 104, no. 12 (2017): 1032–1038. 29173977 10.1016/j.bulcan.2017.10.006

[9] Y. Cai , N. Cheng , H. Ye , F. Li , P. Song , and W. Tang , “The Current Management of Cholangiocarcinoma: A Comparison of Current Guidelines,” Bioscience Trends 10 (2016): 92–102. 27026485 10.5582/bst.2016.01048

[10] T. Shinagawa , T. Tanaka , H. Nozawa , et al., “Comparison of the Guidelines for Colorectal Cancer in Japan, the USA and Europe,” Annals of Gastroenterological Surgery 2 (2018): 6–12. 29863118 10.1002/ags3.12047 PMC5881304

[11] W. S. Tan , S. Rodney , B. Lamb , M. Feneley , and J. Kelly , “Management of Non‐Muscle Invasive Bladder Cancer: A Comprehensive Analysis of Guidelines From the United States, Europe and Asia,” Cancer Treatment Reviews 47 (2016): 22–31. 27231966 10.1016/j.ctrv.2016.05.002

[12] S. J. Yu , A Concise Review of Updated Guidelines Regarding the Management of Hepatocellular Carcinoma Around the World: 2010‐2016,” *Clinical and Molecular Hepatology* 22 (2016): 7–17. 10.3350/cmh.2016.22.1.7 PMC4825164 27044761

[13] Y. Razvi , S. Chan , T. McFarlane , et al., “ASCO, NCCN, MASCC/ESMO: A Comparison of Antiemetic Guidelines for the Treatment of Chemotherapy‐Induced Nausea and Vomiting in Adult Patients,” Supportive Care in Cancer 27 (2019): 87–95. 30284039 10.1007/s00520-018-4464-y

[14] F. Zagouri , P. Liakou , R. Bartsch , et al., “Discrepancies Between ESMO and NCCN Breast Cancer Guidelines: An Appraisal,” Breast 24 (2015): 513–523. 25818651 10.1016/j.breast.2015.02.031

[15] S. Restaino , C. Paglietti , M. Arcieri , et al., “Management of Patients Diagnosed With Endometrial Cancer: Comparison of Guidelines,” Cancers 15, no. 4 (2023): 1091. 36831434 10.3390/cancers15041091 PMC9954548

[16] WHO , Handbook for Guideline Development , 2nd ed. (WHO, 2014).

[17] S. Woolf , H. J. Schünemann , M. P. Eccles , J. M. Grimshaw , and P. Shekelle , “Developing Clinical Practice Guidelines: Types of Evidence and Outcomes; Values and Economics, Synthesis, Grading, and Presentation and Deriving Recommendations,” Implementation Science 7 (2012): 61. 22762158 10.1186/1748-5908-7-61 PMC3436711

[18] B. Fervers , J. S. Burgers , R. Voellinger , et al., “Guideline Adaptation: An Approach to Enhance Efficiency in Guideline Development and Improve Utilisation,” BMJ Quality & Safety 20, no. 3 (2011): 228–236. 10.1136/bmjqs.2010.043257 21209134

[19] B. S. Alper , A. Price , E. J. van Zuuren , et al., “Consistency of Recommendations for Evaluation and Management of Hypertension,” JAMA Network Open 2, no. 11 (2019): e1915975. 31755945 10.1001/jamanetworkopen.2019.15975 PMC6902818

[20] P. Song , Y. Cai , H. Tang , C. Li , and J. Huang , “The Clinical Management of Hepatocellular Carcinoma Worldwide: A Concise Review and Comparison of Current Guidelines From 2001 to 2017,” Bioscience Trends 11 (2017): 389–398. 28904327 10.5582/bst.2017.01202

[21] B. S. Alper , “Making Guidelines Computable,” Clinical and Public Health Guidelines 1 (2024): e12014.

[22] M. P. Hendriks , X. A. A. M. Verbeek , T. van Vegchel , et al., “Transformation of the National Breast Cancer Guideline Into Data‐Driven Clinical Decision Trees,” JCO Clinical Cancer Informatics 3 (2019): 1–14. 10.1200/CCI.18.00150 PMC7101250 31141422

[23] IKNL , ALERTNESS Project (IKNL, 2021), https://iknl.nl/en/guidelines/alertness;-structural-signaling-for-up-to-date-gui.

[24] NVOG and DSfOG , Richtlijn Endometriumcarcinoom (NVOG, 2022), https://richtlijnendatabase.nl/richtlijn/endometriumcarcinoom/startpagina_-_endometriumcarcinoom.html.

[25] NCCN GPUN , Clinical Practice Guideline Uterine Neoplasma (NCCN GPUN, 2025), https://www.nccn.org/professionals/physician_gls/pdf/uterine.pdf.

[26] A. N. Makam and O. K. Nguyen , “An Evidence‐Based Medicine Approach to Antihyperglycemic Therapy in Diabetes Mellitus to Overcome Overtreatment,” Circulation 135, no. 2 (2017): 180–195. 28069712 10.1161/CIRCULATIONAHA.116.022622 PMC5502688

[27] L. Rokach and O. Maimon , eds., “Decision Trees,” in The Data Mining and Knowledge Discovery Handbook (Springer, 2006), 165–192.

