# Supplementary material for: A Computational Method for Systematic Comparison of Clinical Practice Guidelines
Source: Clin Public Health Guidel. 2026 Apr 23;3(2):e70071. doi: 10.1002/gin2.70071 (PMC13563334; doi:10.1002/gin2.70071)
Supplement: Supplementary file 1 [file GIN2-3-e70071-s001.docx]

**Supplement 2. Textual and SNOMED CT based correlation analysis of clinical practice guideline concepts**

| GOPS | Primitive | NVOG concept name | NCCN concept name | Textual relation | SCT code(s) NVOG concept | SCT code(s) NCCN concept | Subdivision correlation | Comment |
| --- | --- | --- | --- | --- | --- | --- | --- | --- |
|  |  |  |  |  |  |  |  |  |
| Initial diagnosis | | | | | | | | |
|  | CDTs | | | | | | | |
|  |  | Additional diagnosis | Additional diagnosis | Identical match | NA | NA | NA |  |
|  |  | Initial diagnosis | Initial evaluation | Correlated match | NA | NA | NA | Similar content of CDTs |
|  |  | Staging diagnostics |  | No match^1^ | NA | NA | NA |  |
|  |  |  | Initial work-up imaging | No match^2^ | NA | NA | NA |  |
|  |  |  | Additional chest imaging | No match^2^ | NA | NA | NA |  |
|  |  |  | Additional diagnostics for incidental findings | No match^2^ | NA | NA | NA |  |
|  |  |  |  |  |  |  |  |  |
|  | PDCs | | | | | | | |
|  |  | Grade | Grade | Identical match | 371469007 \|Histologic grade of neoplasm (observable entity)\| | 371469007 \|Histologic grade of neoplasm (observable entity)\| | NA |  |
|  |  | Ultrasound result |  | No match^1^ | 370380004 \|Ultrasound scan finding (finding)\| |  | NA |  |
|  |  | Endometrial sampling result |  | No match^1^ | No SNOMED CT code |  | NA |  |
|  |  | Suspicion of extension |  | No match^1^ | No SNOMED CT code |  | NA |  |
|  |  |  | Fertility preservation | No match^2^ |  | 702596005 \| Fertility preservation procedure (procedure) \| | NA |  |
|  |  |  | MRI contraindication | No match^2^ |  | No SNOMED CT code | NA |  |
|  |  |  | Type of diagnosis | No match^2^ |  | No SNOMED CT code | NA |  |
|  |  |  | Chest imaging performed | No match^2^ |  | 413815006 \|Chest imaging (procedure)\| | NA |  |
|  |  |  | Suspected metastasis | No match^2^ |  | No SNOMED CT code | NA |  |
|  |  |  | Abnormality seen | No match^2^ |  | No SNOMED CT code | NA |  |
|  |  |  | Morphology | No match^2^ |  | 116676008 \| Associated morphology (attribute) \| | NA |  |
|  |  |  | cT-stage | No match^2^ |  | 399504009 \|cT category (observable entity)\| | NA |  |
|  |  |  |  |  |  |  |  |  |
|  | Interventions | | | | | | | |
|  |  | Transvaginal ultrasound | Transvaginal ultrasound | Identical match | 13205008 \|Transvaginal echography (procedure)\| | 13205008 \|Transvaginal echography (procedure)\| | NA |  |
|  |  | Chest x-ray | Chest x-ray | Identical match | 415232002 \|Radiographic procedure of chest (procedure)\| | 415232002 \|Radiographic procedure of chest (procedure)\| | NA |  |
|  |  | CA-125 | CA-125 | Identical match | 80529009 \|CA 125 measurement (procedure)\| | 80529009 \|CA 125 measurement (procedure)\| | NA |  |
|  |  | Chest/abdominal/pelvic CT | Chest/abdominal/pelvic CT | Identical match | 418023006 \|Computed tomography of chest, abdomen and pelvis (procedure)\| | 418023006 \|Computed tomography of chest, abdomen and pelvis (procedure)\| | NA |  |
|  |  | Pelvic MRI | Pelvic MRI | Identical match | 2690005 \|Magnetic resonance imaging of pelvis (procedure)\| | 2690005 \|Magnetic resonance imaging of pelvis (procedure)\| | NA |  |
|  |  | CT-abdomen | Neck/chest/abdomen/pelvis/groin PET/CT | Correlated match | 169070004 \|Computed tomography of abdomen (procedure)\| | No SNOMED CT code | Correlated NOS | Missing codes |
|  |  | CT-abdomen | Imaging | Correlated match | 169070004 \|Computed tomography of abdomen (procedure)\| | 363679005 \|Imaging (procedure)\| | Strongly correlated |  |
|  |  | Anamnesis |  | No match^1^ | 108217004 \|Interview, history AND/OR physical examination (procedure)\| |  | NA |  |
|  |  | Gynecologic physical examination |  | No match^1^ | 83607001 \|Gynecologic examination (procedure)\| |  | NA |  |
|  |  | Endometrial sampling |  | No match^1^ | 386802000 \|Endometrial biopsy (procedure)\| |  | NA |  |
|  |  | Endocervical curretage |  | No match^1^ | 52889002 \|Endocervical curettage (procedure)\| |  | NA |  |
|  |  | Individualize treatment |  | No match^1^ | 370819000 \|Identification of individual values and wishes concerning care (procedure)\| |  | NA |  |
|  |  | Cystoscopy |  | No match^1^ | 142505009 \|Cystoscopy (procedure)\| |  | NA |  |
|  |  | Rectoscopy |  | No match^1^ | No SNOMED CT code |  | NA |  |
|  |  |  | Chemistry profile | No match^2^ |  | 166312007 \|Blood chemistry (procedure)\| | NA |  |
|  |  |  | Other initial imaging | No match^2^ |  | No SNOMED CT code | NA |  |
|  |  |  | No other imaging | No match^2^ |  | No SNOMED CT code | NA |  |
|  |  |  | Chest CT without contrast | No match^2^ |  | 395081000119108 \|Computed tomography of chest without contrast (procedure)\| | NA |  |
|  |  |  | Adjuvant treatment | No match^2^ |  | No SNOMED CT code | NA |  |
|  |  |  | Additional treatment for high-risk carcinoma | No match^2^ |  | No SNOMED CT code | NA |  |
|  |  |  | No recommendation necessary | No match^2^ |  | No SNOMED CT code | NA |  |
|  |  |  | Cervical biopsy | No match^2^ |  | 50796003 \|Cervical biopsy (procedure)\| | NA |  |
|  |  |  |  |  |  |  |  |  |
|  |  |  |  |  |  |  |  |  |
| Initial treatment | | | | | | | | |
|  | CDTs | | | | | | | |
|  |  | Primary treatment | Primary treatment | Identical match | NA | NA | NA |  |
|  |  | Staging evaluation | Staging evaluation | Identical match | NA | NA | NA |  |
|  |  | Adjuvant treatment endometrioid adenocarcinoma | Adjuvant treatment endometrioid adenocarcinoma | Identical match | NA | NA | NA |  |
|  |  | Adjuvant treatment sereus/clear cell carcinoma | Adjuvant treatment high risk carcinoma | Correlated match | NA | NA | NA | Overlap in population of interest |
|  |  | Childwish and continued treatment | Protocol after endometrial evaluation (fertility sparing) every 3 months | Correlated match | NA | NA | NA | Different perspective, with similar consequences |
|  |  | Policy after hormonal therapy |  | No match^1^ | NA | NA | NA |  |
|  |  | Primary treatment; fertility preserving |  | No match^1^ | NA | NA | NA |  |
|  |  | Conception; fertility preserving |  | No match^1^ | NA | NA | NA |  |
|  |  |  | Re-evaluation for surgery endometrioid carcinoma | No match^2^ | NA | NA | NA |  |
|  |  |  | Protocol after incomplete staging | No match^2^ | NA | NA | NA |  |
|  |  |  | Evaluation of imaging after incomplete staging | No match^2^ | NA | NA | NA |  |
|  |  |  | Re-evaluation for surgery high risk carcinoma | No match^2^ | NA | NA | NA |  |
|  |  |  | Protocol after endometrial evaluation (fertility sparing) every 6 months | No match^2^ | NA | NA | NA |  |
|  |  |  |  |  |  |  |  |  |
|  | PDCs | | | | | | | |
|  |  | Clinical stage | Clinical stage | Identical match | 385349001 \| Clinical stage (observable entity) \| | 385349001 \| Clinical stage (observable entity) \| | NA |  |
|  |  | Grade | Grade | Identical match | 371469007 \|Histologic grade of neoplasm (observable entity)\| | 371469007 \|Histologic grade of neoplasm (observable entity)\| | NA |  |
|  |  | FIGO stage | FIGO stage | Identical match | 385373009 \|International Federation of Gynecology and Obstetrics uterine tumor stage (observable entity)\| | 385373009 \|International Federation of Gynecology and Obstetrics uterine tumor stage (observable entity)\| | NA |  |
|  |  | Lymphovascular space invasion (LVSI) status | LVSI status | Identical match | 371512006 \| Presence of direct invasion by primary malignant neoplasm to lymphatic vessel and/or small blood vessel (observable entity) \| | 371512006 \| Presence of direct invasion by primary malignant neoplasm to lymphatic vessel and/or small blood vessel (observable entity) \| | NA |  |
|  |  | Age | Age | Identical match | 424144002 \| Current chronological age (observable entity) \| | 424144002 \| Current chronological age (observable entity) \| | NA |  |
|  |  | Pathological result | Pathological result | Identical match | 250537006 \|Histopathology finding (finding)\| | 250537006 \|Histopathology finding (finding)\| | NA |  |
|  |  | Staging result | Staging result | Identical match | 399390009 \|Tumor-node-metastasis (TNM) stage grouping (observable entity)\| | 399390009 \|Tumor-node-metastasis (TNM) stage grouping (observable entity)\| | NA |  |
|  |  | Histology | Morphology | Correlated match | No SNOMED CT code | 72724002 \| Morphologic finding (finding) \| | Correlated NOS | Missing codes |
|  |  | Radical treatment possible | Suitable for primary surgery | Correlated match | No SNOMED CT code | 713671003 \|Medically fit for surgery (finding)\| | Correlated NOS | Missing codes |
|  |  | Child wish | Fertility preservation | Correlated match | No SNOMED CT code | 702596005 \| Fertility preservation procedure (procedure) \| | Correlated NOS | Missing codes |
|  |  | Progestron receptor status |  | No match^1^ | 445029000 \| Presence of progesterone receptor in neoplasm (observable entity) \| |  | NA |  |
|  |  | Tumor location |  | No match^1^ | 399687005 \| Anatomic location of primary malignant neoplasm (observable entity) \| |  | NA |  |
|  |  | Time after start treatment |  | No match^1^ | No SNOMED CT code |  | NA |  |
|  |  |  | Contraindication to medical therapy | No match^2^ |  | No SNOMED CT code | NA |  |
|  |  |  | Contraindication to pregnancy | No match^2^ |  | No SNOMED CT code | NA |  |
|  |  |  | Result pregnancy test | No match^2^ |  | 250421003 \| Pregnancy test finding (finding) \| | NA |  |
|  |  |  | Clear demonstration of cervical involvement | No match^2^ |  | No SNOMED CT code | NA |  |
|  |  |  | Presence of extrauterine disease | No match^2^ |  | 1155804009 \|Anatomic location directly invaded by primary malignant neoplasm of uterus (observable entity)\| | NA |  |
|  |  |  | Re-evaluation for surgical resection | No match^2^ |  | No SNOMED CT code | NA |  |
|  |  |  | Response to systemic therapy | No match^2^ |  | 405177001 \| Medication response (observable entity) \| | NA |  |
|  |  |  | Myometrial invasion | No match^2^ |  | 396308005 \| Myometrial invasion by tumor present (finding) \| | NA |  |
|  |  |  | Result imaging | No match^2^ |  | 365853002 \| Imaging finding (finding) \| | NA |  |
|  |  |  | Microscopic cervical invasion | No match^2^ |  | No SNOMED CT code | NA |  |
|  |  |  | Tumor behaviour | No match^2^ |  | No SNOMED CT code | NA |  |

|  |  |  | Previous treatment | No match^2^ |  | No SNOMED CT code | NA |  |
| --- | --- | --- | --- | --- | --- | --- | --- | --- |

|  |  |  | Response to progestin-based therapy at 3-6 months | No match^2^ |  | No SNOMED CT code | NA |  |
| --- | --- | --- | --- | --- | --- | --- | --- | --- |
|  |  |  | Response | No match^2^ |  | 102468005 \| Therapeutic response, function (observable entity) \| | NA |  |
|  |  |  | Childbearing complete | No match^2^ |  | No SNOMED CT code | NA |  |
|  |  |  | Pregnant | No match^2^ |  | 77386006 \| Pregnancy (finding) \| | NA |  |
|  |  |  | Actively trying to conceive | No match^2^ |  | 169449001 \|Trying to conceive (finding)\| | NA |  |
|  |  |  |  |  |  |  |  |  |
|  | Interventions | | | | | | | |
|  |  | Bilateral salpingo oophorectomy | Bilateral salpingo oophorectomy | Identical match | 29827000 \|Excision of bilateral fallopian tubes and ovaries (procedure)\| | 29827000 \|Excision of bilateral fallopian tubes and ovaries (procedure)\| | NA |  |
|  |  | EBRT | EBRT | Identical match | 33195004 \|External beam radiation therapy procedure (procedure)\| | 33195004 \|External beam radiation therapy procedure (procedure)\| | NA |  |
|  |  | Brachytherapy | Brachytherapy | Identical match | 152198000 \|Brachytherapy (procedure)\| | 152198000 \|Brachytherapy (procedure)\| | NA |  |
|  |  | Complete staging | Complete staging | Identical match | No SNOMED CT code | No SNOMED CT code |  |  |
|  |  | Re-staging | Re-staging | Identical match | No SNOMED CT code | No SNOMED CT code |  |  |
|  |  | Radiotherapy | Radiotherapy | Identical match | 1287742003 \|Radiotherapy (procedure)\| | 1287742003 \|Radiotherapy (procedure)\| |  |  |
|  |  | Debulking | Debulking | Identical match | 238302003 \|Debulking of pelvic tumor (procedure)\| | 238302003 \|Debulking of pelvic tumor (procedure)\| |  |  |
|  |  | Vaginal brachytherapy | Vaginal brachytherapy | Identical match | 311887001 \|Insertion of vaginal cesium applicators (procedure)\| | 311887001 \|Insertion of vaginal cesium applicators (procedure)\| |  |  |
|  |  | Endometrial sampling | Endometrial sampling | Identical match | 386802000 \|Endometrial biopsy (procedure)\| | 386802000 \|Endometrial biopsy (procedure)\| |  |  |
|  |  | Adjuvant treatment | Adjuvant treatment | Identical match | No SNOMED CT code | No SNOMED CT code |  | Missing codes |
|  |  | Start conception | Start conception | Identical match | 711038007 \| Promotion of conception (procedure) \| | 711038007 \| Promotion of conception (procedure) \| |  |  |
|  |  | MRI | Imaging | Correlated match | 113091000 \|Magnetic resonance imaging (procedure)\| | 363679005 \|Imaging (procedure)\| | Moderately correlated |  |
|  |  | Total abdominal hysterectomy | Total hysterectomy | Correlated match | 116143008 \|Total abdominal hysterectomy (procedure)\| | 116140006 \|Total hysterectomy (procedure)\| | Strongly correlated |  |
|  |  | Total laporoscopic hysterectomy | Total hysterectomy | Correlated match | 236888001 \|Laparoscopic total hysterectomy (procedure)\| | 116140006 \|Total hysterectomy (procedure)\| | Strongly correlated |  |
|  |  | Hysterectomy | Radical hysterectomy | Correlated match | 236886002 \|Hysterectomy (procedure)\| | 116142003 \|Radical hysterectomy (procedure)\| | Moderately correlated |  |
|  |  | Hysterectomy | Surgery | Correlated match | 236886002 \|Hysterectomy (procedure)\| | 387713003 \|Surgical procedure (procedure)\| | Moderately correlated |  |
|  |  | Medroxyprogesteron | Systemic therapy | Correlated match | No SNOMED CT code | No SNOMED CT code | Correlated NOS | Missing codes |
|  |  | Megestrol acetate | Systemic therapy | Correlated match | No SNOMED CT code | No SNOMED CT code | Correlated NOS | Missing codes |
|  |  | Chemotherapy | Systemic therapy | Correlated match | 367336001 \|Chemotherapy (procedure)\| | No SNOMED CT code | Correlated NOS | Missing codes |
|  |  | Individualize treatment | Observe | Correlated match | 370819000 \|Identification of individual values and wishes concerning care (procedure)\| | No SNOMED CT code | Correlated NOS | Missing codes |
|  |  | Tamoxifen | Hormone therapy | Correlated match | 724161009 \|Tamoxifen therapy (procedure)\| | 169413002 \|Hormone therapy (procedure)\| | Moderately correlated |  |
|  |  | Endometrial sampling | Endometrial evaluation | Correlated match | 386802000 \|Endometrial biopsy (procedure)\| | No SNOMED CT code | Correlated NOS | Missing codes |
|  |  | Hormonal containing UID | Continuous progestin-based therapy | Correlated match | 472837007 \|Insertion of hormone releasing intrauterine contraceptive device\| | No SNOMED CT code | Correlated NOS | Missing codes |
|  |  | Hormonal containing UID | Maintenance progestin-based therapy | Correlated match | 472837007 \|Insertion of hormone releasing intrauterine contraceptive device\| | No SNOMED CT code | Correlated NOS | Missing codes |
|  |  | Hysteroscopy |  | No match^1^ | 233545006 \|Hysteroscopy (procedure)\| |  | NA |  |
|  |  | Referral to oncological center |  | No match^1^ | No SNOMED CT code |  | NA |  |
|  |  | Resection lymph node metastases |  | No match^1^ | 24223006 \|Excision of lesion of lymph structure (procedure)\| |  | NA |  |
|  |  | Standard treatment |  | No match^1^ | 372921003 \|Providing care according to standard (regime/therapy)\| |  | NA |  |
|  |  |  | Counseling fertility sparing option | No match^2^ |  | 702595009 \|Counseling for fertility preservation (procedure)\| | NA |  |
|  |  |  | Consultation with fertility expert | No match^2^ |  | No SNOMED CT code | NA |  |
|  |  |  | Genetic counseling | No match^2^ |  | 79841006 \|Genetic counseling (procedure)\| | NA |  |
|  |  |  | Genetic testing | No match^2^ |  | 405824009 \|Genetic test (procedure)\| | NA |  |
|  |  |  | Weight management | No match^2^ |  | 718361005 \|Weight management program (regime/therapy)\| | NA |  |
|  |  |  | Stereotactic body radiotherapy | No match^2^ |  | 395096001 \|Stereotactic radiotherapy (procedure)\| | NA |  |
|  |  |  | Surgical resection | No match^2^ |  | 65801008 \|Excision (procedure)\| | NA |  |
|  |  |  | Surveillance | No match^2^ |  | 225419007 \|Surveillance (regime/therapy)\| | NA |  |
|  |  |  | Best supportive care | No match^2^ |  | 243114000 \|Support (regime/therapy)\| | NA |  |
|  |  |  | Pathological confirmation of metastatic disease | No match^2^ |  | No SNOMED CT code | NA |  |
|  |  |  | Re-evaluate for surgical resection | No match^2^ |  | No SNOMED CT code | NA |  |
|  |  |  | Surgical staging | No match^2^ |  | No SNOMED CT code | NA |  |
|  |  |  |  |  |  |  |  |  |
|  |  |  |  |  |  |  |  |  |
| Follow-up | | | | | | | | |
|  | CDTs | | | | | | | |
|  |  | Follow-up | Surveillance | Correlated match | NA | NA | NA |  |
|  |  | Follow-up; during conception phase |  | No match^1^ | NA | NA | NA |  |
|  |  | Continuation follow-up; during conception phase |  | No match^1^ | NA | NA | NA |  |
|  |  |  |  |  |  |  |  |  |
|  | PDCs | | | | | | | |
|  |  | Years since ending primary treatment | Years since ending primary treatment | Identical match | No SNOMED CT code | No SNOMED CT code | NA | Missing codes |
|  |  | Pregnancy |  | No match^1^ | 77386006 \| Pregnancy (finding) \| |  | NA |  |
|  |  | Child wish |  | No match^1^ | No SNOMED CT code |  | NA |  |
|  |  | Cytology result |  | No match^1^ | 365716004 \| Finding of cytology - general (finding) \| |  | NA |  |
|  |  |  |  |  |  |  |  |  |
|  | Interventions | | | | | | | |
|  |  | Physical examination | Physical examination | Identical match | 5880005 \|Physical examination procedure (procedure)\| | 5880005 \|Physical examination procedure (procedure)\| | NA |  |
|  |  | Ultrasound | Imaging | Correlated match | 16310003 \|Ultrasonography (procedure)\| | 363679005 \|Imaging (procedure)\| | Moderately correlated |  |
|  |  | Gynecologic physical examination |  | No match^1^ | 83607001 \|Gynecologic examination (procedure)\| |  | NA |  |
|  |  | Consultation |  | No match^1^ | 11429006 \|Consultation (procedure)\| |  | NA |  |
|  |  | Taking vaginatop cytology |  | No match^1^ | 438633009 \|Sampling of vaginal vault for smear (procedure)\| |  | NA |  |
|  |  | Pipelle |  | No match^1^ | 134279003 \|Endometrial sampling using pipelle (procedure)\| |  | NA |  |
|  |  | Hysteroscopy |  | No match^1^ | 233545006 \|Hysteroscopy (procedure)\| |  | NA |  |
|  |  | Standard treatment |  | No match^1^ | 372921003 \|Providing care according to standard (regime/therapy)\| |  | NA |  |
|  |  | Follow-up |  | No match^1^ | 390906007 \|Follow-up encounter (procedure)\| |  | NA |  |
|  |  |  | CA-125 | No match^2^ |  | 80529009 \|CA 125 measurement (procedure)\| | NA |  |
|  |  |  | Patient education | No match^2^ |  | 311401005 \|Patient education (procedure)\| | NA |  |
|  |  |  |  |  |  |  |  |  |
| Recurrence diagnostics | | | | | | | | |
|  | CDTs | | | | | | | |
|  |  | Recurrence diagnostics | Recurrence diagnostics | Identical match | NA | NA | NA |  |
|  |  |  |  |  |  |  |  |  |
|  | PDCs | | | | | | | |
|  |  | Type of recurrence | Suspected recurrence | Correlated match | No SNOMED CT code | No SNOMED CT code | Correlated NOS | Missing codes |
|  |  |  |  |  |  |  |  |  |
|  | Interventions | | | | | | | |
|  |  | CT-thorax+abdomen | Abdominal+pelvic CT | Correlated match | 418891003 \|Computed tomography of chest and abdomen (procedure)\| | 419394006 \|Computed tomography of abdomen and pelvis (procedure)\| | Strongly correlated |  |
|  |  | CT-thorax+abdomen | Chest CT | Correlated match | 418891003 \|Computed tomography of chest and abdomen (procedure)\| | 169069000 \|Computed tomography of chest (procedure)\| | Strongly correlated |  |
|  |  | CT-thorax+abdomen | Abdominal+pelvic MRI | Correlated match | 418891003 \|Computed tomography of chest and abdomen (procedure)\| | 419150008 \|Magnetic resonance imaging of abdomen and pelvis (procedure)\| | Strongly correlated |  |
|  |  | CT-thorax+abdomen | Imaging | Correlated match | 418891003 \|Computed tomography of chest and abdomen (procedure)\| | 363679005 \|Imaging (procedure)\| | Moderately correlated |  |
|  |  | Bone scan | Whole body PET+CT | Correlated match | 41747008 \|Radioisotope scan of bone (procedure)\| | 480621000119104 \|Positron emission tomography with computed tomography of whole body (procedure)\| | Moderately correlated |  |
|  |  | Bone scan | Imaging | Correlated match | 41747008 \|Radioisotope scan of bone (procedure)\| | 363679005 \|Imaging (procedure)\| | Moderately correlated |  |
|  |  | Biopsy |  | No match^1^ | 86273004 \|Biopsy (procedure)\| |  | NA |  |
|  |  | Bood cell count |  | No match^1^ | 88308000 \|Blood cell count (procedure)\| |  | NA |  |
|  |  | CA-125 |  | No match^1^ | 80529009 \|CA 125 measurement (procedure)\| |  | NA |  |
|  |  | Cystoscopy |  | No match^1^ | 142505009 \|Cystoscopy (procedure)\| |  | NA |  |
|  |  | Rectoscopy |  | No match^1^ | No SNOMED CT code |  | NA |  |
|  |  |  |  |  |  |  |  |  |
|  |  |  |  |  |  |  |  |  |
| Recurrence treatment | | | | | | | | |
|  | CDTs | | | | | | | |
|  |  | Recurrence treatment | Therapy for relapse | Correlated match | NA | NA | NA |  |
|  |  | Policy after hormonal therapy |  | No match^1^ | NA | NA | NA |  |
|  |  |  | Additional therapy for locoregioal relapse | No match^2^ | NA | NA | NA |  |
|  |  |  | Protocol after therapy for relapse | No match^2^ | NA | NA | NA |  |
|  |  |  |  |  |  |  |  |  |
|  | PDCs | | | | | | | |
|  |  | Type of recurrence | Recurrence type | Correlated match | No SNOMED CT code | No SNOMED CT code | Correlated NOS | Missing codes |
|  |  | Prior treatment | Initial radiation therapy to site of recurrence | Correlated match | No SNOMED CT code | No SNOMED CT code | Correlated NOS | Missing codes |
|  |  | Prior treatment | Kind of previous radiotherapy | Correlated match | No SNOMED CT code | No SNOMED CT code | Correlated NOS | Missing codes |
|  |  | Recurrence size | Number of metastases | Correlated match | No SNOMED CT code | 252105006 \| Number of metastases (observable entity) \| | Correlated NOS | Missing codes |
|  |  | Location regional recurrence | Disease location | Correlated match | No SNOMED CT code | 371480007 \| Anatomic location of neoplasm (observable entity) \| | Correlated NOS | Missing codes |
|  |  | Treatment possibility |  | No match^1^ | No SNOMED CT code |  | NA |  |
|  |  | Progesteron receptor status |  | No match^1^ | 445029000 \| Presence of progesterone receptor in neoplasm (observable entity) \| |  | NA |  |
|  |  |  | Type distant metastases | No match^2^ |  | No SNOMED CT code | NA |  |
|  |  |  | Locoregional recurrence | No match^2^ |  | No SNOMED CT code | NA |  |
|  |  |  | Residual disease | No match^2^ |  | 1295428008 \| Unintended postoperative presence of residual neoplasm following excision of neoplasm (finding) \| | NA |  |
|  |  |  | Disease status | No match^2^ |  | No SNOMED CT code | NA |  |
|  |  |  |  |  |  |  |  |  |
|  | Interventions | | | | | | | |
|  |  | Radiotherapy | EBRT | Correlated match | 1287742003 \|Radiotherapy (procedure)\| | 33195004 \|External beam radiation therapy procedure (procedure)\| | Moderately correlated |  |
|  |  | Radiotherapy | Brachytherapy | Correlated match | 1287742003 \|Radiotherapy (procedure)\| | 152198000 \|Brachytherapy (procedure)\| | Moderately correlated |  |
|  |  | Radiotherapy | IORT | Correlated match | 1287742003 \|Radiotherapy (procedure)\| | 168524008 \|Radiotherapy - intraoperative control (procedure)\| | Moderately correlated |  |
|  |  | Radiotherapy | Ablative radiotherapy | Correlated match | 1287742003 \|Radiotherapy (procedure)\| | No SNOMED CT code | Correlated NOS | Missing codes |
|  |  | Radiotherapy | Palliative EBRT | Correlated match | 1287742003 \|Radiotherapy (procedure)\| | No SNOMED CT code | Correlated NOS | Missing codes |
|  |  | Debulking | Surgical exploration | Correlated match | 238302003 \|Debulking of pelvic tumor (procedure)\| | 76145000 \|Exploratory incision (procedure)\| | Moderately correlated |  |
|  |  | Debulking | Resection | Correlated match | 238302003 \|Debulking of pelvic tumor (procedure)\| | 65801008 \|Excision (procedure)\| | Moderately correlated |  |
|  |  | Debulking | Surgical resection of distant metastases | Correlated match | 238302003 \|Debulking of pelvic tumor (procedure)\| | No SNOMED CT code | Correlated NOS | Missing codes |
|  |  | Exenteration | Surgical exploration | Correlated match | 14509009 \|Evisceration (procedure)\| | 76145000 \|Exploratory incision (procedure)\| | Moderately correlated |  |
|  |  | Exenteration | Resection | Correlated match | 14509009 \|Evisceration (procedure)\| | 65801008 \|Excision (procedure)\| | Moderately correlated |  |
|  |  | Exenteration | Surgical resection of distant metastases | Correlated match | 14509009 \|Evisceration (procedure)\| | No SNOMED CT code | Correlated NOS | Missing codes |
|  |  | Hormone therapy | Systemic therapy | Correlated match | 169413002 \|Hormone therapy (procedure)\| | No SNOMED CT code | Correlated NOS | Missing codes |
|  |  | Systemic chemotherapy | Systemic therapy | Correlated match | No SNOMED CT code | No SNOMED CT code | Correlated NOS | Missing codes |
|  |  | Medroxyprogestron | Systemic therapy | Correlated match | No SNOMED CT code | No SNOMED CT code | Correlated NOS | Missing codes |
|  |  | Megestrolacetate | Systemic therapy | Correlated match | No SNOMED CT code | No SNOMED CT code | Correlated NOS | Missing codes |
|  |  | Tamoxifen | Systemic therapy | Correlated match | 724161009 \|Tamoxifen therapy (procedure)\| | No SNOMED CT code | Correlated NOS | Missing codes |
|  |  | Refer to ongological center |  | No match^1^ | No SNOMED CT code |  | NA |  |
|  |  | Continue surveillance |  | No match^2^ |  | 225419007 \|Surveillance (regime/therapy)\| | NA |  |
|  |  | Best supportive care |  | No match^2^ |  | 243114000 \|Support (regime/therapy)\| | NA |  |

*Attachment 1. For each step (Initial diagnosis, Initial treatment, Follow-up, Recurrence diagnosis, and Recurrence treatment) in the generic oncological process (GOPS), the primitives (clinical decision trees (CDTs), patient and disease characteristics (PDCs), and interventions) from both guidelines were compared at the semantic level. Relations were categorized as ‘exact match’, ‘correlated match’, ‘no match1’ (present in NVOG only), or ‘no match2’ (present in NCCN only). Additionally, PDC and Intervention concepts from NVOG and NCCN that were classified as ‘exact match’ or ‘correlated match’ on semantic comparison were also compared using the terminology system Systemized Nomenclature of Medicine – Clinical Terms (SNOMED CT). In SNOMED CT concepts are logically defined by their relationships. A descriptive form of the concept definition can be derived from hierarchical relationships and defining attribute relationships. The comparison results in the following relation categories: ‘Identical’, ‘Hierarchical’ (parent/child relation), ‘Attribute value’ (characteristic of the meaning), and ‘Hierarchical and attribute value’. NA = Not Applicable.*
