# Supplementary material for: A Computational Method for Systematic Comparison of Clinical Practice Guidelines
Source: Clin Public Health Guidel. 2026 Apr 23;3(2):e70071. doi: 10.1002/gin2.70071 (PMC13563334; doi:10.1002/gin2.70071)
Supplement: Supplementary file 2 [file GIN2-3-e70071-s002.docx]

**Supplement 1.**

**Refinement of the Applied Methodology in Attachment 2.**

To systematically compare the clinical decision trees (CDTs), patient and disease characteristics (PDCs), and interventions outlined in both guidelines, we conducted a semantic-level analysis. Each step in the oncological treatment process was examined to determine the relation between concepts.

Concepts were categorized based on textual comparison into three primary groups:

- **Identical Match** – Concepts were identical in meaning.
- **Correlated Match** – Concepts were related but not identical.
- **No Match** – Concepts were present in only one of the two guidelines.

To refine the classification, we applied SNOMED CT, a standardized medical terminology system, to identify the varying degrees of correlation. This approach ensured a structured and consistent evaluation, leading to the following classification:

1. **Identical Match** – Concepts were exact synonyms.
   - *Example:* The term *grade* appeared in both guidelines with identical meaning.
2. **Correlated Match** – Concepts were related but not identical, further divided into:
   - **Strongly Correlated** – Concepts shared both hierarchical and attribute relationships.
     - *Example:* *CT abdomen* and *PET-CT neck/chest/abdomen/pelvis*. Both involve computed tomography (CT), with overlapping procedural attributes, but PET-CT includes additional imaging techniques and anatomical regions.
   - **Moderately Correlated** – Concepts had a clear hierarchical connection.
     - *Example:* *General imaging* vs. *MRI*. Both refer to imaging, but MRI represents a more specific modality.
   - **Weakly Correlated** – Concepts were related through attributes but lacked a strong hierarchical link.
     - *Example:* Endometrial sampling vs. Endometrial evaluation. Endometrial sampling and endometrial evaluation, are both procedures involve an intervention on the endometrium. Sampling is a specific method that might contribute to evaluation, but the two concepts are not hierarchically related.
3. **Correlated Not Otherwise Specified (NOS)** – Concepts appeared correlated based on textual assessment, but concepts were not represented in SNOMED CT and therefore no further analysis was done. SNOMED CT enables more in-depth analysis through its logic-based structure and the use of postcoordination, as outlined in the editorial guide. However, accurate modeling of terms requires substantial research and input from guideline developers. Based on our textual analysis, these concepts were classified as *Correlated NOS*.
4. **No Match** – Concepts were exclusive to one guideline.
   - *Example:* A recommendation found in the NVOG guideline but absent from the NCCN guideline, or vice versa.

**Concept Definitions and Hierarchies**

Within SNOMED CT, each concept is defined by a fully specified name (FSN) and linked to related concepts through hierarchical and attribute relationships. Concepts classified as defined have sufficient attributes to distinguish them from similar terms, while primitive concepts lack a complete definition.

- **Interventions** were mapped to the procedure hierarchy. When pharmaceutical substances were involved, they were standardized as:
  - *Administration of [substance] (procedure)*
  - *[Substance] therapy (therapy)*
  - *[Substance] regimen (regimen)*
- **Medications.** When SNOMED CT codes related to the administration of pharmaceutical substances were available, an in-depth analysis was conducted using the substance and pharmaceutical product hierarchies. This allowed for grouping of medications based on shared pharmacological properties—for example, "has disposition: progesterone receptor agonist.”
- **Patient and disease characteristics** were primarily mapped to the clinical finding hierarchy but also integrated with other relevant hierarchies:
  - *Situation with explicit context* – Applied when characteristics involved a clinical status (e.g., suspected vs. confirmed).
  - *Procedure hierarchy* – Used for prior interventions to ensure comparability.

Further refinements were made using attributes from the SNOMED CT Model Component module, but only attributes relevant to clinical findings, observable entities, procedures, or contextual situations were included.

**Methodological Considerations**

To ensure accuracy and consistency in concept mapping, a certified SNOMED CT terminologist participated in the evaluation. The February 2024 international edition of SNOMED CT served as the standard reference.

However, some limitations remained:

- Certain concepts could not be fully defined within the SNOMED CT framework, limiting precise comparison.
- Some concepts from one guideline corresponded to multiple concepts in the other guideline, complicating direct one-to-one mapping.

**Conclusion**

By applying a structured classification system and leveraging SNOMED CT for refinement, this methodology enhances guideline comparability and interoperability. The results highlight key semantic similarities and discrepancies between guidelines, providing insights that can support harmonization efforts, clinical decision-support systems, and improved healthcare interoperability.
